# Application of and Elements of Success in Crown Work

**Published:** 1886-09

**Authors:** C. L. Anderson

**Affiliations:** Washington, D. C.


### APPLICATION OF AND ELEMENTS OF SUCCESS IN CROWN WORK.

BY C. L. ANDERSON, D. D. S., WASHINGTON, D. C.

READ BEFORE THE CONNECTICUT VALLEY DENTAL ASSOCIATION, AT HARTFORD, JUNE 11, 1886.

In the practice of such a comparatively new work as is the crown system, the exposition of which has not been general or thorough while the desire to practice it has been universal, it is not surprising that the proportion of failures has been so large, for the few who have made of it a specialty have been developers of a work, rather than practitioners of an exact demonstration.

That crown work of varying styles has been occasionally made by operators here and there is not denied, and that it is absolutely new in the strict interpretation of the term is not claimed. But the introduction of the Richmond Crown and its modification marked the point where the profession took another long stride in enlarging its usefulness, and a nearer approach to preserving the dental arch in normal beauty and form. The immediate recognition the work received illustrates how ready the profession is to adopt and execute. The introduction of a large number of rival crowns is a striking indication that the practice is sound, and the endeavor now is to attain to a greater degree of perfection in the application of the principle. But experience, I believe, has proved beyond a doubt that, as a rule, a root or tooth must be capped to permanently preserve from decay and insure stability of the attached crown. The band-crowns are the only ones which can be used on all teeth and roots on either jaw, under any arrangement of teeth, form of jaw, or demand of function. Other crowns can be used in certain cases, but are not capable of such universal application. That this work, however constructed, will be a failure in the hands of many, needs no demonstration. The desire for an acquirement of speed at the expense of skillful manipulation, is responsible for the larger part of these misfits.

No work that we now attempt needs as much care in detail and such thoroughness in manipulation. Being in so large a degree a mechanical operation, it demands for success exactness, and a concert-pitch effort each time.

The preparation of the roots or teeth is the first consideration.

All the exposed portions of tooth should be beveled carefully, so that the largest diameter shall be at the gum line, and no band should extend beneath the free margin of the gum tissue more than $\frac{1}{32}$ of an inch. Failure to observe these points will result in pockets from ill-fitting bands, absorption of gum and alveolar tissue and pericemental membrane, with consequent evil results. As regards incisors, and usually all other roots for porcelain-faced crowns, as much of the tooth structure should be preserved, with the exception of the labial or buccal wall, as is consistent with a proper occlusion, and avoidance of undue bulk. This will tend to prevent the temptation to drive the band deep to secure a grip. After proper beveling, a burr or Sheffield scaler should be used, especially with the incisors, to remove the enamel at the neck of the tooth and to secure such a form of the root as to insure the fitting of the band, no matter how tapering the root may be. By thus limiting the width of the band and making a form which can be fitted easily, a large factor in failures will be eliminated, for the many have attempted to fit roots as found, which is usually impossible. When the canals need enlarging, by driving in a plug of soft wood dipped in creosote, which can be easily drilled, it will serve as a guide and prevent the troublesome hole through the apical foramen at the side of the root. I have found that the use of a hand drill to obtain the depth desired, and a subsequent enlarging with the engine burr, obviates these surprising disclosures, as well as the painful and damaging degree of heat often accompanying the use of the engine. Having fitted and trimmed the band with careful reference to the form of the gum and alveolus, so that it extends an even depth in every portion, the proper bevel is secured for the labial or buccal face. A single piece of metal can be bent and soldered so as to make a closed cap, allowing the porcelain to occupy any position desired, and necessitating the use of cement, not for securing in position the crown or bridge, but simply to exclude moisture from the cap and roots. By using a rather large piece of solder and simply sweating the band together, the top can be soldered without the use of extra material, making a stronger cap, and one which will fit snugly to the root because of the absence of surplus solder in the cap.

The fitting of a porcelain face to a single crown can be done nicely in the mouth, if time permit, but when a number of crowns or bridges are to be made, the taking of a bite and a plaster impression with caps in position, articulating as for plate-work, will secure greater accuracy and speed. By allowing the pins to extend as far out of the caps as the occlusion will permit of, having the exposed portion file-notched, they will be withdrawn with the impression, and the proper position maintained, so that the fit of the caps is not injured, and they can be soldered with the remainder of the case. It may be stated as a rule in bridge-work, that a case will go on with comparative ease, when in removing the impression the caps have remained firm in the plaster. Care should be exercised in preparing the roots and fitting caps to have them parallel.

A surplus of wax in taking a bite should be avoided, that a proper position on the caps and model may be secured. The use of a model of the teeth of the opposing jaw, with or without the wax bite according to the occlusion, gives still better results than wax alone. A proper occlusion of crowns or bridges is absolutely essential to permanency. By the use of cusps on the crowns, and by striking up surfaces for mastication from zinc dies made from models of the natural teeth, and soldering these surfaces to each dummy crown, it is as easy to get as perfect an occlusion of a bridge posterior to the cuspids, as with plain teeth in plate work. The surfaces should be filled with small balls of metal on the under side, to add strength and solidity and to avoid burning them in soldering. Very little solder should be used in attaching the surfaces to the teeth, so that wires of platina-iridium can be fitted without making too much bulk or exposure in finishing. The incisors should be soldered with a surplus for short bites, and ground to an occlusion in the mouth. In other cases, gold shoulders similar to cusps for gold crowns should be fitted and soldered with the final heat. The lengthening of crowned teeth and bridges, and of the opposing teeth, is very frequent, due to a lack of care in these particulars. In many cases of side bridges, an impression of the masticating surfaces of the opposing teeth can be secured, and a continuous surface swaged up and fitted after the porcelains are in position on the articulator. It should be waxed firmly in position and the edges burnished to the teeth nicely, to prevent pockets. The wax used for this work, on account of its tenacity, is prepared as follows: White wax, seven parts, gum damar four parts, and resin a little, according to the season, using more in summer. The crown-metal manufactured for the writer stands so high a degree of heat, even at No. 32 thickness, that it is highly recommended, being inconspicuous in color and extra strong. (To avoid the appearance of advertising, the name of the manufacturer is withheld, except on application.) Care, in the instances noted, will secure the results named, and we may make crowns and bridge-work which will be permanent, for irritation from bands is avoided, and with proper occlusions exercise is insured, and this will maintain health.

The breaking of porcelains is urged as an objection to this work in bridges. This can be entirely obviated, and without the display of gold often seen on side bridges. After the porcelains have been ground into position and backed up, bevel the cutting edges as much as possible without changing the form, even if it be a cuspid or bicuspid face, and wax carefully on the bevel a piece of crown metal, No. 25 to 27, according to the strain, taking care to keep the wax from the joint, and allowing the metal to extend beyond the tooth, so as to be held firmly in position by the investment when the wax is removed, and while soldering. If the cutting edges of the incisors occlude, flatten and bevel slightly. When finely finished the protection will not be observed, and I have yet to see the first case of breakage in an extensive use of this simple device. In protecting bicuspid and molar faces, the protection must be secured in position before the masticating surfaces are fitted, which should be as near the cutting edge as the occlusion will permit. If properly finished, no gold will show, and the porcelain beveled in this manner is thoroughly protected from strain. Avoid getting wax on the face of the metal, so that the investment may protect it from solder and the porcelain from borax checks, compelling the solder to attach underneath, so that the face will need no finishing, except to grind down the portion beyond the tooth. It will also allow of a high polish for the surface, and there will be no thinning of the protection from careless grinding, as there would be if solder were allowed to flow on the face, and the tendency to discoloration at the cutting edge will be avoided.

An objection urged against this work is the formation of abscesses. It is understood that disease should be cured before attaching crowns. If the pulp canals are properly treated and filled before the insertion, and each occlusion is secured, there is little liability to diseased action. It is this ability to secure an occlusion that makes the use of a crown desirable, and obviates the necessity of long, tedious operations in plugging, which, after all, amount as a rule to nothing more than replacing in part lost tissue without restoring to the teeth needed exercise by proper occlusion. Extensive contouring should be displaced in practice by fine crowns, for the benefit of both patient and operator. Crowning is not liable to be followed by periosteal irritation from the thousands of blows necessary to condense the precious metal.

To produce permanent bridge-work, the length of the teeth inserted should always be less than the original crowns, for the object is to decrease the leverage and strain, and this can be properly done, for this style of work of any magnitude is rarely undertaken except in persons past middle life, when the teeth are normally shorter than the full crown of youth. Care in this respect, and a proper occlusion that the strain may be distributed, and never opposing in the form of inclined planes, will not cause loosening or irritation. But the tying of teeth together is to be done with judgment, for as a rule the fewer teeth so united the better. Short bridges are successes, when the same united together might be failures. In full cases, four or six teeth in proper position are preferable to a greater number of attachments. Bridge-work, properly made, allows of less strain to the individual tooth than it ever has, except in a full arch of natural teeth. But the conditions so vary that it would be folly to attempt to recommend any definite rules, except the exercise of sound judgment and careful manipulation. When the writer sees the superior incisors supported by the canines, and the inferior molars and perhaps bicuspids lacking and no artificial substitute for them, and other cases constructed under similar conditions, he is not surprised that thoughtless ones judge the whole work a sham, and cry fraud. Operative dentistry, judged by the efforts of men like these, would earn no better reputation.

Granting that the objections presented have been, as we believe, successfully overcome, the monster stumbling block of uncleanliness lies in our path. No work in the mouth, natural or artificial, is or can be absolutely clean. Pass a silk between the teeth, and is the result pleasing to your olfactory sense? Then what is expected is comparative cleanliness, so that the lack of perfection in this respect will not be detected by others or ourselves, except on severe test. But the claim is made, backed up by reason and experience, that bridge-work properly made and adjusted is capable of being kept free from uncleanliness easier than the natural teeth. In criticism, the same rules that apply to the natural teeth should apply to bridge-work. Because patients neglect their natural teeth, allow stain and deposits to accumulate, we do not advise removing the teeth, but thorough cleansing. So with this work.

To begin, the parts of a bridge should be perfectly fitted and soldered, and when finished present a continuous smooth surface entire. If there are pits from excessive use of borax, pockets, crevices, etc., the case should be finished and put through the fire again. The greatest care should be exercised to have the metal portion of the bridge entirely free from the gum tissue, so that a soft brush can without difficulty reach every portion of it. The porcelains should stand off from the gum, so that water can be used to clear out saliva or occasional lodgment of food. By coating the cast with thick shellac varnish before grinding the porcelains in position, the proper distance will be secured. It is only in the anterior part of the mouth, where the form of the ridge will permit of it and the perfection of speech demands it, that the porcelains should pass by the ridge to any considerable extent. Care in the continuity of surface and form of the bridge, so that the bevel from the masticating surface to the porcelain shoulder shall be regular and without pockets, will produce bridges, (and my experience is uniform on this point,) that can be kept perfectly clean, and with less effort than would be demanded to secure the same results with the natural teeth. But if there is any doubt on this point, is there any reason why the patient should not have the bridge cleaned and polished once in six months or a year, and is there any doubt of his ability to thoroughly cleanse it if properly made? The advantages of this work are so apparent that the slight disadvantage of having to take care of it is hardly worth consideration. The majority of bridges may undoubtedly have been improperly formed and defectively and carelessly finished, so that it is no wonder they have nearly overcome us with their stench, but our effort should be, in view of the great benefit derived from properly constructed bridges, to endeavor to span this cess pool of primitive bridge-work to the solid accomplishment of what we know can and should be done. The successful application of the crown system, when all the conditions above are observed and the work constructed as indicated, will depend upon the conscientious judgment of the operator. Its real domain is the restoration of teeth literally worn out, when we already have a good foundation, and the restoration at the time of life indicated will be permanent. If bridge-work is adjusted much before middle life, the conditions will, in all probability, so change that its permanency must be considered doubtful, and very little should be done, except that which is absolutely demanded to preserve teeth until the time for permanent work has arrived. The indiscriminate use of bridge-work as a practice can result in nothing but failure. The strain on roots is greater in young persons as the leverage is stronger, and while exceptional cases may prove successful, the real use and place of this work is the restoration of mouths to proper appearance and function when the probability of change of condition is not a factor.

For patients whose teeth are chalky, brittle, or exceptionally prone to decay, it would seem much better practice to preserve each tooth by careful crowning, than to repeatedly fill and finally lose valuable teeth, while there is much less pain and expense to the patient. Such work brings to the operator a class of teeth which he can handle with far more success than is now generally attained.

Another cause of failure in crown work is the attaching of two or more dummy crowns to a single support. An incisor root can, if care is used to prevent direct strain on the dummy, carry a single tooth, but never more, and no root posterior to the canine should ever be expected to support the strain of mastication on more surfaces than its own crown presents. Such and similar devices only serve to bring discredit on a system, which, if properly practiced, would produce beautiful and permanent results.

In such cases as it may seem desirable or necessary to remove at the will of the patient, the use of movable bridges can be resorted to, and this branch of crown work is susceptible of indefinite modification to meet the varying requirements presented. In several cases where the patients were inveterate chewers of tobacco, and whose mouths indicated unusual neglect as regards cleanliness, the writer has made movable bridges which have proved, in trials of from six months to over two years use, a most gratifying success, in both upper and lower jaws. The construction of these cases requires still more time and care than the stationary bridge, with special attention to the strength of each part, to avoid change in removing, cleansing and readjusting. The use of movable appliances involves the preparation of the teeth or roots as for ordinary crown work, and the capping of them with thinner metal than usual, say No. 36, as there is no strain on the caps, and bulk is to be avoided. Where pins are to be used, a piece of the metal can be fitted to the squared pin-wire and soldered neatly on the outside, making a square tube, which should be closed at the apical end, and the length having been ascertained, it is placed in position and soldered to the hole made in the cap for it, and the cap and tube thus united thoroughly cemented to the root. In constructing large cases of bridge-work of any style, as few pins as possible should be used, so that the bridge may be adjusted with as little strain as may be. Excessive drilling of the roots should be avoided. Few bridges require more than two pins, and many only one, especially when gold crowns are included, and there is a good occlusion on the sides. The practice of putting a pin in each root involves more work, with far less nicety of accomplishment. When all the caps are nicely fitted, the use of so many pins only results in cutting away teeth and roots to get the case on, ruining what otherwise might be a perfect operation. I have a case of six inferior incisors and a first bicuspid, single crowns, on roots in all of which are live pulps, allowing only the neat beveling and fitting of caps, and the use of porcelains, so that no gold is exposed. This I did three years ago, and upon examination since beginning this paper, although they have had all the work of mastication owing to loss of the remaining natural teeth, they are as healthy and strong as when the work was done. If single crowns will stand this strain, bridges with caps as carefully fitted and with as proper an occlusion, should need no extra pins. In movable bridges, where the squared pin is to fit into a metal tube as closely as possible, two should be the limit, and dependence should be placed on the proper construction of the other fastenings, or second caps. To go into the details of the work would extend this paper beyond my time or present opportunity. The only purpose is to indicate how the work is done, and then special instruction should follow. This will avoid much costly and disappointing experimentation on valuable patients.

Due care in the use of unnecessary pins will stop the unwarrantable, sinful destruction of pulps. I have put as high as fifteen crowns in one mouth, the incisors and bicuspid crowns porcelain-faced, and preserved the pulp in each tooth. When pulps are to be removed, it can be done with little or no pain by obtunding according to the conditions. But arsenic should not be used, or the liability of chronic ulceration, or gradual breaking down of the pericemental membrane will result.

When teeth or roots have slightly loosened from lack of or a mal-occlusion, a restoration to correct conditions by the use of crowns has been followed by happy results. The restoration of exercise, or the proper application of the strain is commendable, but those whom the worship of the golden calf has transferred into fit companions for the long-eared quadruped, and who place crowns and bridges on roots suffering from pyorrhœa, chronic ulceration and absorption of root tissue, and on roots without the possibility of occlusion, bring discredit on this work, as they do on all other operations requiring skill and the conscientious use of judgment, and it is no wonder many exclaim "uneasy lies the head that wears a crown." As a rule, all dental operations are performed too hurriedly, and when criticism is made and greater care and a nicer class of work recommended, the general excuse is that "people won't pay for it." I believe from observation that he who constantly gives to his patients his very best efforts, and always persists in demanding their acceptance of the best styles of work, will find that his only difficulty will be to meet the demand for his services. A reputation of this kind takes time to develop, but no capital yields such rich, certain and constant dividends.

When for any reason a clasp plate is to be worn, by capping the teeth to which the clasp would be fitted the permanency of the operation is assured, the teeth protected from wear, a better fit and grip of clasps and a smaller plate made possible, and the patient saved the annoyance of sensitive surfaces, decay, and loss of valuable teeth. By a proper apposition of the edges of the band before soldering, all crowns will have the largest diameter just below the masticating surface. But when used for clasping, the bulging should be marked and regular, otherwise in time a slight dropping of the plate might follow from continued use, and a slipping on and off of the clasps.

Crowns will also be found of great value in correcting irregularities, when the age of the patient or the character of the case does not permit of changing the position of the tooth or teeth by the usual appliances.

Much has been said and written about the effect of pulpless teeth remaining in the jaw. While my practice, as indicated, is not to destroy except when absolutely necessary (and with continued experience the necessity diminishes), yet the results have been so satisfactory when the pulp has been properly removed, and I have seen so many cases where pulpless teeth have done good service from ten to thirty-five years, and still remain in good condition, that I believe that with proper support and occlusion annoyance will be the rare exception rather than the rule.

Filling of teeth is decidedly a temporary operation compared with crown work, either in live or pulpless teeth, and must remain so until our researches shall have developed a practice of prevention of decay, rather than remedying the effects of it by fillings which are decently permanent only in cases of the most favorable description. As long as this is a fact, the grafting of indestructible crowns on roots perfectly protected from decay, or the capping of partially destroyed crowns likewise protected, will, if faithfully practiced, give to our patients sets of teeth which will forever protect them from the pain and discomfort of plates, preserve the expression of the face and function of the mouth, and save many from the torments of dyspepsia and lack of ability to properly masticate or enjoy food.

With a recognition of the value of this work our success will often be a fact, where now our best efforts are apologies, and it will enable the careful mechanic-dentist to produce work which in appearance and use cannot be rivalled by the devotee of tedious gilded contouring. It will do away with the resort to unsightly, incomplete operations with the amalgams of the "new departurists," who have not the skill or wisdom to hide their tarnished efforts. There is real merit in both horns of this dental dilemma, but the adoption of the crown work will serve to prevent being gored by either of them. The crown is truly the "golden mean." Its adoption is not a radical step, but at this time is eminently conservative. In closing, I am reminded of a couplet often quoted, but apropos in this connection :

> Be not the first by whom the new is tried;
> Or yet the last to lay the old aside.

